# Engineering metabolite-responsive transcriptional factors to sense small molecules in eukaryotes: current state and perspectives

**DOI:** 10.1186/s12934-019-1111-3

**Published:** 2019-03-26

**Authors:** Xia Wan, Monireh Marsafari, Peng Xu

**Affiliations:** 1 Department of Chemical Biochemical and Environmental Engineering, University of Maryland Baltimore County, Baltimore, MD 21250 USA; 2 Oil Crops Research Institute of Chinese Academy of Agricultural Sciences, Wuhan, 430062 Hubei China; 3 Key Laboratory of Biology and Genetic Improvement of Oil Crops, Ministry of Agriculture, Wuhan, 430062 Hubei China; 4 Department of Agronomy and Plant Breeding, University of Guilan, Rasht, Islamic Republic of Iran

**Keywords:** Transcriptional factors, Sensors, Eukaryotic cells, Metabolic engineering, Intelligent biomanufacturing, Synthetic biology

## Abstract

Nature has evolved exquisite sensing mechanisms to detect cellular and environmental signals surrounding living organisms. These biosensors have been widely used to sense small molecules, detect environmental cues and diagnose disease markers. Metabolic engineers and synthetic biologists have been able to exploit metabolites-responsive transcriptional factors (MRTFs) as basic tools to rewire cell metabolism, reprogram cellular activity as well as boost cell’s productivity. This is commonly achieved by integrating sensor-actuator systems with biocatalytic functions and dynamically allocating cellular resources to drive carbon flux toward the target pathway. Up to date, most of identified MRTFs are derived from bacteria. As an endeavor to advance intelligent biomanufacturing in yeast cell factory, we will summarize the opportunities and challenges to transfer the bacteria-derived MRTFs to expand the small-molecule sensing capability in eukaryotic cells. We will discuss the design principles underlying MRTF-based biosensors in eukaryotic cells, including the choice of reliable reporters and the characterization tools to minimize background noise, strategies to tune the sensor dynamic range, sensitivity and specificity, as well as the criteria to engineer activator and repressor-based biosensors. Due to the physical separation of transcription and protein expression in eukaryotes, we argue that nuclear import/export mechanism of MRTFs across the nuclear membrane plays a critical role in regulating the MRTF sensor dynamics. Precisely-controlled MRTF response will allow us to repurpose the vast majority of transcriptional factors as molecular switches to achieve temporal or spatial gene expression in eukaryotes. Uncovering this knowledge will inform us fundamental design principles to deliver robust cell factories and enable the design of reprogrammable and predictable biological systems for intelligent biomanufacturing, smart therapeutics or precision medicine in the foreseeable future.

## Background

Biosensors are indispensable tools to detect or respond to a specific biochemical signal [1–3]. Commonly used biosensors generally fall into two categories: electrochemical biosensors and optical biosensor [4]. The former converts a chemical gradient potential into an electrical signal, the transducer domain of electrochemical biosensor is coupled with electron transfer of an oxidation–reduction reaction mediated through enzyme or non-enzyme catalysis. Common examples of electrochemical biosensor include oxygen probe, CO_2_ probe, glucose sensor and foam sensor (based on conductivity), which have been widely applied in bioprocess engineering and large-scale fermentation [5]. The latter converts a chemical gradient signal into an optical output, either absorbance, fluorescence or luminescence [6]. The transducer domain of optical biosensor relies on various biomolecule interactions that lead to the formation of colorimetric, fluorometric or luminescent molecules. These biomolecule interactions include protein–ligand (which is the case in enzyme–substrate or allosteric interaction) [6, 7], protein–protein (i.e. immunological interaction or GPCR receptor) [8], protein-DNA-RNAP (i.e. transcriptional regulation) [9], RNA–RNA (i.e. riboregulators and toehold switches) [10–13], DNA/RNA-ligand (i.e. aptamers) interactions [14, 15].

**Fig. 1:**
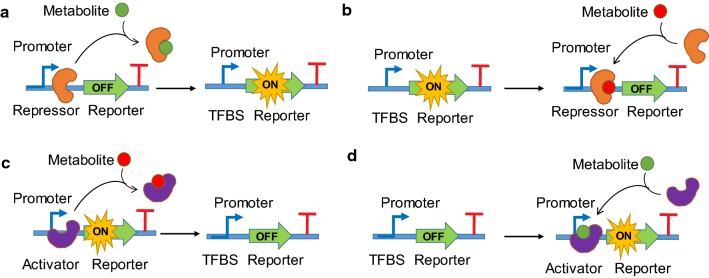
Generalized principles of metabolite-responsible transcriptional factors (MRTFs) in biological systems. **a** Repressor binds with TFBS (typically, an operator) to block RNA polymerase for transcribing the target gene. Metabolite abolishes repression by removing the roadblock. **b** Repressor binds with metabolite (co-repressor) to form an active transcriptional roadblock and prevents transcription. **c** Activator binds with TFBS (typically, an enhancer element) to recruit RNA polymerase for transcribing the target gene. Metabolites abolishes activation by removing the activator. **d** Activator binds with metabolite (co-activator) to form an active transcriptional recruiter and accelerates transcription. TFBS: transcriptional factor binding sites

Transcriptional factor (TF) based biosensors typically consist of a repressor or activator protein regulating the transcriptional activity of a specific promoter. A cis-regulatory DNA sequence (generally called operator or enhancer) adjacent to the promoter is the core DNA element that binds with a TF restricting or enhancing the access of RNA polymerase (RNAP) to the promoter. A repressor binds to the operator and prevents RNAP proceeding forward to decrease transcription (Fig. 1a, b); an activator binds to the enhancer elements and promotes the formation of more stable RNAP-promoter complex to increase transcription (Fig. 1c, d) [16, 17]. Apart from the DNA-binding domain, TFs also contain a ligand-binding domain which is the sensor domain that responds to small molecules or environmental stress signal (salt, osmosis, pH, oxygen, redox, light or radiation etc.).

Repressor or activator protein typically transduces a C-terminal ligand-binding activity to the N-terminal DNA-binding activity. Upon interaction with a small molecule or environmental stress signal, TFs will undergo a conformational change leading to altered binding affinity between RNAP and the regulated promoter. The RNAP is typically designed to drive (actuate) the transcription of a reporter protein that outputs an easily measured optical or biochemical signal (absorbance, fluorescence or luminescence) [18]. In principle, small molecule or environmental stimuli input will form a dose–response correlation with reporter output. In a word, the C-terminal ligand-binding domain of TFs dictates the specificity of the input–output relationship, while the N-terminal DNA-binding domain of TFs dictates the sensitivity of the input–output relationship.

**Fig. 2:**
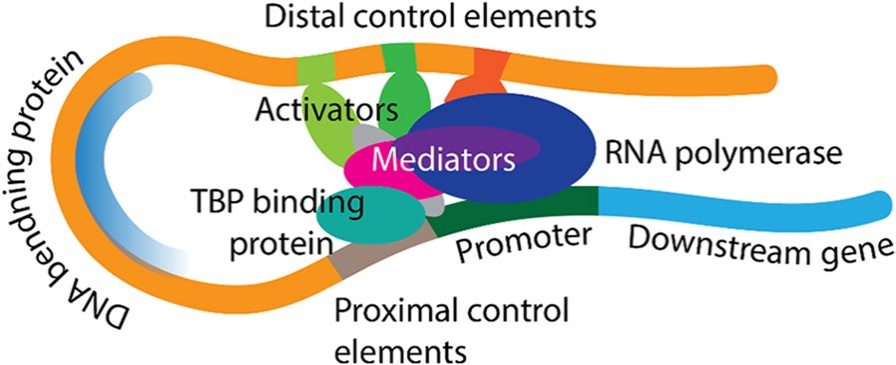
Complex transcriptional factor interactions stabilize transcriptional bubble and recruit RNA polymerase to transcribe the downstream gene in eukaryotes. *TBP* TATA-binding protein

Recent development in metabolite-responsive transcriptional factor (MRTF) based biosensors have expanded our ability to reprogram gene expression or control metabolic activity [19–22]. Most of these biosensors are developed in bacterial system. Eukaryotic gene transcription typically involves many DNA-binding proteins associated together to recruit RNA polymerase, bend/loop the template DNA, and stabilize the transcriptional complex inside the nucleus (Fig. 2). Due to the complexity of transcriptional regulation and the physical barrier of nucleus membrane separating transcription and translation, we argue that the knowledge or the design principles underlying MRTF-based biosensors derived from prokaryotic systems may not be directly translated to eukaryotic system. We will summarize the opportunities and challenges to transfer the bacteria-derived MRTFs to expand the small-molecule sensing capability in eukaryotic cells. We will discuss the design principles underlying MRTF-based biosensors in eukaryotic cells, including the choice of reliable reporters and the characterization tools to minimize background noise, strategies to tune the sensor dynamic range, sensitivity and specificity, as well as the criteria to engineer activator and repressor-based biosensors. Due to the physical separation of transcription and protein expression, we will also summarize the nuclear import/export mechanism of MRTFs across the nuclear membrane. Harnessing this knowledge will inform us fundamental design principles to engineer robust cell factories and enable the design of reprogrammable and predictable biological systems for intelligent biomanufacturing and smart therapeutics.

## The choice of reporter genes and characterization tools in eukaryotes

Classical optical biosensors are primarily converting a chemical signal into a colorimetric output. For instance, blue-white screening is based on the catalytic property of lacZ (β-galactosidase, which cleaves X-gal and releases X chromogenic moiety, thus display blue color), and the expression of lacZ is transcriptionally controlled by inducer IPTG or lactose. The input concentration of the inducer (IPTG or lactose) forms a quantitative correlation with the intensity of the output signal (the blue color), which can be spectrophotometrically measured in the case of β-galactosidase assay. Due to the low sensitivity or unavailability of chromogenic compounds, researchers have turned to develop new chemical entity that could emit fluorescence or luminescence. For example, both the fluorescent substrate (MUG, 4-methylumbelliferyl-β-d-galactopyranoside) and histochemical staining substrate (X-gluc, 5-bromo-4-chloro-3-indolyl-β-d-glucuronic acid) has been used for β-glucuronidase (GUS) assay, but the fluorescence substrate could reach a much higher sensitivity and detection limit than the X-gluc straining assay [35]. Instead of using small fluorogenic molecules, fluorescence proteins have been widely used as reporters due to their strong emission [36, 37], relatively low background noise and easy molecular manipulation (cloning, gene expression and purification). Likewise, firefly (*Photinus pyralis*) or sea pansy (*Renilla reniformis*) luciferase is also a widely used reporter candidate if the other two types of reporter are not readily applicable [38, 39]. These two luciferases differ in their sizes, and substrate and cofactor requirements. *Renilla* luciferase is an ATP-dependent enzyme with 36 kDa molecular weight, while firefly luciferase is ATP-dependent and is a much larger protein (61 kDa). In addition, firefly luciferase generates yellow light ranging from 550 to 570 nm. By contrast, *Renilla* luciferase emits blue light at a wavelength of 480 nm. Therefore, these two luciferases are compatible and can be used as a dual- reporter system.

Besides, a very small luciferase with only 19 kDa from luminous shrimp *Oplophorus gracilirostris* has been improved by mutagenesis and then developed as a promising reporter enzyme named as NanoLuc or Nluc [40]. Nluc has already been widely used in biomedical experiments for investigation of protein–protein or ligand–protein interaction, gene regulation, molecular imaging and photodynamic therapy [41]. Neither ATP nor Mg^2+^ is required for the reaction system. The emission wavelength is 460 nm, which is different from that from firefly or *Renilla* luciferase. It has been well-demonstrated that Nluc exhibits several advantages over other bioluminescence enzymes, including enhanced stability and sensitivity, versatile applications in eukaryotic cells including yeast and mammal cells. However, the unique synthetic substrate furimazine for Nluc seems toxic to mammal cells in vivo and in vitro [42].

Both yeast-enhanced GFP (yEGFP) and luciferase are now two most widely used reporters in eukaryotic biosensor system. Compared to luciferase or Nluc, although no substrate is needed, fluorescence protein requires high-energy excitatory light and the background noise is generally strong. The background noises are typically derived from yeast autofluorescence, metabolic heterogeneity, and light emission or reflection due to internal organelles or thick cell wall. The increasingly complex interior structure of eukaryotic cell demands more sensitive imaging instrument (i.e. fluorescence microscopy or flow cytometry) to scan a population of cell and determine the mean fluorescence Intensity (MFI) of the output signal. In addition, fluorescence proteins are sensitive to pH, metal ions and oxygen levels. For these reasons, new reporter protein, ATP-independent Nanoluc luciferase, has emerged as the promising substitute to probe transient transcriptional activity in eukaryotes. Not to mention the extremely low background signal, luciferase could be also used as whole-cell assay, thanks to the cell-permeable furimazine substrate and the intensive luminescence emission. To minimize leaky/basal expression and eliminate background noise, it is generally an imperative practice to wash the tested cell with PBS saline buffer. A time-course of luciferase reading with different levels of effector molecules could be continuously recorded with a plate reader. Then a dose–response curve between input effector concentration and the output reporter could be established, where dynamic response range, sensitivity and specificity could be determined from the dose–response curve.

## Tuning MRTF dynamic range, sensitivity and specificity in eukaryotes

**Fig. 3:**
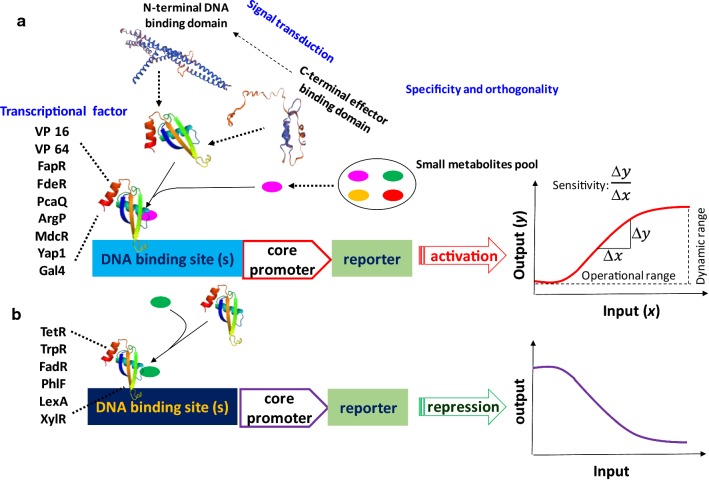
Dissecting the design criteria of engineering metabolite responsive transcriptional factors (MRTFs) in eukaryotic cells. **a** Engineering activator-based MRTF sensors in eukaryotic cells. **b** Engineering repressor-based MRTF sensors in eukaryotic cells. VP 16, VP 64, FapR, FdeR, PcaQ, ArgP, MdcR, Yap1 and Gal4 are a collection of transcriptional activators that are commonly used in eukaryotic cells. TetR, TrpR, FadR, PhlF, LexA and XylR are representative transcriptional repressors commonly used in eukaryotic cells. Core promoter or minimal promoter contain only the core TF binding sites and the TATA box

In addition to the choice of reporter genes, the architecture of the promoter for the MRTF and the reporter gene are also crucial for fine-tuning of both the operational and dynamic ranges. Engineering native promoters seems to be the first choice in most reported studies [27, 33]. However, the promoter strength for MRTFs and the reporter gene should be well-controlled. Strong promoter probably would cause too much background noise or leaky expression even in the absence of the effector molecule (i.e. an inducer). Constitutive or inducible expression of the MRTF will also affect the time-response dynamics of the sensor-actuator input–output relationship. In many cases, there are negative autoregulation or positive autoregulation of the MRTF, namely the expression of MRTF from the native promoter is controlled by the same effector molecules. This autoregulation is a result of the evolution of the basic transcriptional motifs to make cell precisely control transcriptional events and readily adapt to changing environmental conditions. Working with orthogonal transcriptional system, the use of heterologous MRTFs or the construction of hybrid promoters may solve this problem (Fig. 3a). Indeed, in many cases, one should avoid using the native promoter which may be subject to many endogenous regulations. Deletion of upstream activator or repressor binding sites (distal control elements in Fig. 2) of native promoters helps to eliminate or decrease such undesired noise [25–27, 33]. On the contrary, weak native promoter may not allow the abundant expression of the MRTF which compromises the detection limit. As a general practice, a proximal promoter that contains about 200 bp upstream of the transcriptional start site (TSS) should be the ideal length to create hybrid promoter to study combinatorial genetics or engineer MRTF-based biosensors in eukaryotes (Fig. 3a). A medium strength constitutive promoter may be suitable for the expression of the MRTF to prevent any unintended transcriptional events.

Insertion of additional DNA binding sites into native promoter is an alternative for fine-tuning of sensor output (Fig. 3a). The type and the position of inserted MRTF binding sites are both critical to improve the sensor dynamic response. Insertion of such TF-binding sites upstream of TATA sequence are generally acceptable, whereas downstream or flanked to the TATA sequence probably causes adverse effects [43]. Despite that down-regulation of promoter strength are commonly observed, in practice the sensitivity and dynamic range may be improved accordingly [26, 27, 34]. The number of activator or repressor-biding sites to flank the core promoter should not be ignored. The binding of multiple MRTFs to the hybrid promoter may synergistically or gradually change the stability of RNAP-promoter complex, this cooperative binding is always an effective approach to tuning the dynamic transcriptional response and the sensitivity of the engineered biosensors.

Lots of native promoters are transcriptionally responsive to media components. Catabolite repression plays a critical role, especially high glucose concentration, nitrogen starvation or the presence of stress signals may shift the transcriptional dynamics. One would desire to find promoters that are minimally affected by environmental conditions. In terms of consistent transcriptional output, one should also consider chromosomal integration of the sensor construct into the genome, instead of detecting sensor activity from episomal plasmid. Engineering a reliable biosensor with the right dynamic response range, sensitivity and specificity is a challenging task. It would require lots of trial-and-error testing before a predictable and sensitive sensor construct could be obtained.

## Engineering transcriptional activator-based biosensor in eukaryotes

Eukaryotic cells are more complex than prokaryotic cells in terms of transcriptional regulation. In eukaryotic cells, activator along with chromatin-modifying enzymes and basal transcription factors work together to activate gene expression in a precise manner [44]. Transcriptional activator enhances gene expression by increasing the accessibility of transcriptional machinery (a protein complex that mediates the signals between activator and RNA polymerase II) to bind the transcription start site and initiate the transcription (Fig. 2) [45]. There is no such transcriptional machinery found in bacteria. Representative activators and their functional mechanism have been well summarized [22]. Of them, a Herpes simplex virus transcriptional activator VP16 can activate transcriptional initiation of many eukaryotic genes (Fig. 3a) and thus is widely used in many eukaryotic biosensor models [24–26].

In most of the designs, bacterial transcriptional activators can be directly applied into eukaryotic cells (Table 1). Sigma factors are essential to prokaryotic transcriptional initiation. However, sigma factor or other auxiliary transcriptional enzyme seems not crucial when prokaryotic transcriptional activators are directly transferred into eukaryotic cells (Table 1). The utilization of bacterial-derived instead of endogenous transcriptional activators (eTA) would avoid the cross-talk effect that these eTAs may exert on other native promoters. In a recent example, the prokaryote superfamily of LysR-type transcriptional regulators (LTTRs) have been engineered to construct sensors that are responsive to their cognate small-molecule inducers [46]. The engineered biosensors have been used for in vivo screening of naringenin, muonic acid [46] and itaconic acid [47] with a quantitative dose-dependent manner.

**Table 1:** Representative examples and design principles of engineering metabolite-responsive transcriptional factors (TFs) in eukaryotes

Host	TF and source	Effector (small metabolite)	Reporter	Transcriptional effect	Characteristic/architecture
Mammalian cells					
Hela cells	TetR-VP16	Doxycycline		Transcription activator	TetR blocks the transcription of tetA, which encoding for the tetracycline efflux pump. When tetracycline binds to TetR, tetA would be expressed and functioning as tetracycline export pump [23]
COS-1 cells	FapR-VP16	Malonyl-CoA	Luciferase or a destabilized short half-life GFP	Transcriptional activator	*Bacillus subtilis* FapR as a transcriptional repressor inhibits most of genes involved in fatty acid biosynthesis. FapR undergoes a conformation shift when malonyl-CoA binding to it and thus releases operators of many fatty acid synthesis genes. While VP16 is a herpes virus transcriptional activator. In frame fusion of FapR with VP16 converts FapR into a transcriptional activator in the absence of malonyl-CoA. Nucleus localization signal (NLS) is used [24]
Human embryonic kidney (HEK293) and Chinese hamster ovary (CHO) cells	*E.coli* PhlF, or PhlF-VP16	2,4-Diacetylphloroglucinol	YFP	Transcriptional repressor (PhlF) or Transcriptional activator (PhlF-VP16)	PhlF is equipped with eukaryotic specific nuclear localization signal (NLS). And multiple operator sites are integrated into responsive promoters [25]
Human K562 cells	VP16	Digoxin or progesterone	yEGFP	Transcriptional activator	The same biosensor as used in yeast [26]
Human K562 cells	none	none	EGFP	CRISPR/Cas9 genome editing	Ligand biding domain DIG_3_ and PRO1 were fused upstream of a non-functional EGF variant with a premature stop codon. A gRNA was designed to target premature stop codon and to restore the EGFP activity [26]
Yeast					
*Saccharomyces cerevisiae*	LysR-type transcriptional regulator BenM from *Acinetobacter* sp. ADP1	*Cis,cis*-muconic acid (CCM)	Green fluorescence protein (GFP)	Transcription activator	DNA-binding site of BenM (BenO) was inserted into a truncated CYC1 promoter [27]
*S. cerevisiae*	FdeR from *Herbaspirillum seropedicae*	Naringenin	GFP	Transcription activator	DNA-binding site of BenM (BenO) was inserted into a truncated CYC1 promoter [27]
*S. cerevisiae*	PcaQ from *Sinorhizobium meliloti*	Protocatechuic acid	GFP	Transcription activator	DNA-binding site of BenM (BenO) was inserted into a truncated CYC1 promoter [27]
*S. cerevisiae*	ArgP from *Escherichia coli*	l-Arginine	GFP	Transcription activator	DNA-binding site of BenM (BenO) was inserted into a truncated CYC1 promoter [27]
*S. cerevisiae*	MdcR from *Klebsiella pneumonia*	Malonic acid	GFP	Transcription activator	DNA-binding site of BenM (BenO) was inserted into a truncated CYC1 promoter [27]
*S. cerevisiae*	Tetracycline-responsive TetR	Tetracycline		Transcription repressor	Hybrid TetO-CYC promoter [28]
*S. cerevisiae*	FadR *from Escherichia coli* *and* *Vibrio cholerae*	Fatty acid or fatty acyl-CoA	yEGFP	Transcription activator	bacterial FadR transcriptional repressors and yeast synthetic promoters containing varying number of FadR‐binding operators [29]
*S. cerevisiae*	MetJ-B42	S-adenosyl-methionine	Venus, *HIS3*	Transcriptional activator	Transcription factor domain B42 is fused with *E. coli* MetJ [30]
*S. cerevisiae*	XylR from *Tetragenococcus halophile, Clostridium difficile*, and *Lactobacillus pentosus*	Xylose sugars	yEGFP	Transcription repressor	Constitutive expression of heterologous XylR under a synthetic promoter with XylR operator-binding sites [31]
*S. cerevisiae*	Gal4-Ada	Methyl phosphotriester adduct	GFP	Transcriptional activation	Fusing the N-terminal domain of *E. coli* Ada protein, which can detect methylating compounds, to the Gal4 transcriptional activator [32]
*S. cerevisiae*	FapR from *B. subtilis*	Malonyl-CoA	GFP	Transcription activator	Malonyl-CoA reductase (MCRCa) from *Chloroflexus aurantiacus* is under the control of FapR, to create a self-regulatory system [33]
*S. cerevisiae*	Yap1 from *S. cerevisiae*	Diamide	GFP	Transcription activator	Yap1 target promoter TRX2 with an extra yap responsive stie, or TRX2 promoter is fused with 1-5 upstream activating sequence [34]
*S. cerevisiae*	The herpes virus protein VP16 or VP64	Digoxin	yEGFP	Transcription activator	Computationally-designed ligand binding domain DIG_0_ or PRO_0_ was inserted between N-terminal DNA binding domain and C-terminal transcriptional activation domain [26]
*S. cerevisiae*	LexA	Digoxin	luciferase	Transcription repressor	Replace the Gal4 DNA binding sites in *GAL1* promoter with LexA binding sites [26]
Plant					
*Arabidopsis thaliana*	The herpes virus protein VP16	Digoxin or digaxigenin	Luciferase	Transcription activator	A degron MATα2 from *Arabidopsis* with VP16 transcriptional activation domain were inserted downstream of a Gal4-activated plant promoter [26]

Of interest, the bacterial transcriptional repressor can also be converted into a transcriptional activator and used in eukaryotic cells. FapR from *B. subtilis* is a transcriptional repressor that inhibits many genes involved in fatty acid synthesis. However, when FapR is in-frame fused with a transcriptional activator VP16 to form FapR-VP16, which turns FapR into a transcriptional activator in the absence of malonyl-CoA. By contrast, with increased levels of malonyl-CoA, FapR would dimerize and abolishe the activation function of VP16 [24]. This strategy has been successfully used to engineer a genetically-encoded malonyl-CoA sensor in human cells. A similar strategy is also demonstrated by transferring transcriptional repressor TetR into a transcriptional activator in mammal cells [25].

DNA binding domains (DBD) of MRTFs are generally conserved among the same family of TFs. It enables us to find DBD from new species or de novo design the DBD [22]. With the large volume of high-resolution protein structures, we will be able to design and engineer novel MRTFs with improved DNA binding affinity (Fig. 3a). It is possible to design nucleotides sequence-specific DNA binding domains with the help of Zinc-finger DNA binding domain, basic leucine zipper domain (bZIP domain), TALEN DNA-binding domain, and CRISPR-dCas9-gRNA ribonucleoprotein complex. The DNA binding domain of these artificial TFs could be configured to fuse with an effector binding domain (EBD), where the binding of a small molecule with EBD would alter the conformation of DBD, leading to the dissociation of the DBD from its cognate DNA binding sites. As a result, the accessibility and stability of RNAP-promoter complex will change and exhibit differential transcriptional output. This may provide a general strategy to convert a metabolite-binding protein into a MRTF. In a recent study, a programmable DNA binding motif (zinc finger module) with a maltose binding protein have been welded together to generate a novel biosensor conferring maltose-regulated gene expression [21].

Specific ligand binding domain (LBD) can also be rationally designed. Modular biosensor could be constructed by fusing a conditionally destabilized LBD with a reporter or a transcriptional activator [26]. Binding of the ligand will stabilize the sensor-reporter/regulator complex and lead to enhanced reporter activity. For example, a rationally designed ligand binding scaffold (DIG0) was inserted between the N-terminal DNA-binding domain and C-terminal transcriptional activation domain of VP16 or VP64. This artificial MRTF has been successfully used to sense digoxin and progesterone in yeast, human cell or plant, as well as to improve the biosynthetic yield of progesterone [26].

## Engineering transcriptional repressor-based biosensor in eukaryotes

Due to the complexity of eukaryotic transcriptional regulation, the repressor-operator ribonucleoprotein complex (i.e. LacI–LacO complex in lac operon) that represses bacterial transcription is not the only transcriptional repression mechanism in eukaryotes. Instead, the repressor prevents the transcription via multiple mechanisms in eukaryotic cells. Generally, eukaryotic transcriptional repression can be classified into three categories: inhibition/destabilization of the basal transcriptional machinery, deactivation of activator and remodeling of chromatin or nucleosome 3-D structure [48].

Despite of these multiple repression mechanisms, bacteria-derived transcriptional repressors have been successfully engineered as biosensors in yeast and mammalian cells [23]. Three representative examples of transcriptional repressors-based biosensors in eukaryotic cells are summarized in Table 1. For example, *B. subtilis* transcriptional repressor *Fap*R along its cognate DNA-binding site *fap*O has been engineered to construct a malonyl-CoA sensor in *S. cerevisiae*. This malonyl-CoA sensor was used to screen a genome-wide overexpression library, and the authors identified promising gene targets to improve intracellular malonyl-CoA and 3-hydroxypropionic acid production in Bakers’ yeast [33]. In a similar study, bacterial repressor has been used to construct a xylose‐sensing genetic circuit in *S. cerevisiae*. By tuning the repressor expression, operator position and operator sequence, the authors improved the induction ratio (dynamic response range) and sensitivity with defined dose–response relationship [49].

The amount of repressor protein present in the cell seems to be a critical factor determining the functionality of bacteria-derived MRTFs in yeast (Fig. 3b). This was best illustrated by the construction of the modular fatty acid/fatty acyl‐CoA biosensor in *S. cerevisiae* [29]. Bacterial *Fad*R repressor was engineered to suppress the transcriptional activity of a yeast synthetic promoter containing *Fad*R‐binding operator. It was observed that only overexpression of FadR under the strong and constitutive *TEF1* promoter can trigger the repression of reporter gene [29]. Although a weaker promoter would lead to a shift in dynamic range, strong promoter for MRTFs is still the favorable choice due to the stronger repression effect. Accordingly, varying the promoter strength and the number of *Fad*R-binding sites in the synthetic promoter were found as general strategies to tune the sensor activity.

Nuclease-deficient CRISPR system (dCas9) is also designed to repress gene expression in many eukaryotic cells, however, the efficiency is much lower compared with applications in prokaryotic cells [50]. It is predicted that a single dCas9-sgRNA complex may not effectively stop transcription initiation. Based on this hypothesis, transcriptional repressor domain could be fused with dCas9 to enhance the repression effect [51, 52]. The repressor domain, theoretically, should bind its cognate operator region and bring the dCas9-sgRNA complex in close proximity to the promoter, thereby block transcription more efficiently. It is most likely that the operator-repressor-dCas9-sgRNA-promoter ribonucleoprotein complex will form a more stable roadblock to prevent the recruitment of RNA polymerase. Of tested repression domains, a mammalian transcriptional repressor domain Mxi, which interacts with histone deacetylase to change nucleosome occupancy states, exhibits strong synergistic repression effect with dCas9 [51]. Such dCas9-Mxi system along with multiplex sgRNA have been used to suppress nonhomologous end-joining (NHEJ) and promote homology-directed recombination (HDR) in *Y. lipolytica* [53].

Regardless transcriptional activation or repression, mutagenesis might be an effective strategy to obtain ligand binding domain with higher affinity and specificity. In light of not-fully-characterized effector binding domain, de novo design of small molecule-binding domain is also a promising approach [26]. Computational de novo design of ligand binding domain (LBD) and protein evolution should pave the way to facilitate the design and engineering of artificial MRTFs with improved sensing capability.

## Nuclear import and export of transcriptionally-active MRTFs

Unlike prokaryotic cells, most transcriptional regulation takes place in nucleus in eukaryotic cells. To enable precise gene expression control, the cells have developed mechanism to import and accumulate TFs in nucleus. Theoretically, macromolecules with 60 kDa or below would be freely diffused into nucleus via nuclear pore complex (NPC), which is embedded in the nuclear envelope [54, 55]. Otherwise, assembly of a nucleus localization signal (NLS) into the biosensor architecture is necessary. SV40 from simian virus is a universal NLS and it has been successfully applied in many yeast or mammalian cell biosensors [33, 56].

The existence of nucleus compartment and membrane effectively separates transcriptional regulation with protein expression, as a result, a NLS is generally required to lead the MRTFs through the nucleus envelope. On the other hand, it is not always the case that the small metabolites or effector molecules are freely permeable to the nucleus membrane. The switch between low (OFF) and high (ON) transcriptional activity require that the transcriptionally active MRTFs should be selectively imported into nucleus and interact with the target promoters to control gene expression. The selective partition of the transcriptionally-active MRTFs into the nucleus necessitates us to understand how basic TFs are imported to the nucleus. Harnessing this knowledge will inform us fundamental design principles to effectively engineer MRTF-based sensors, improve the sensitivity and dynamic response range of MRTFs in eukaryotes.

**Fig. 4:**
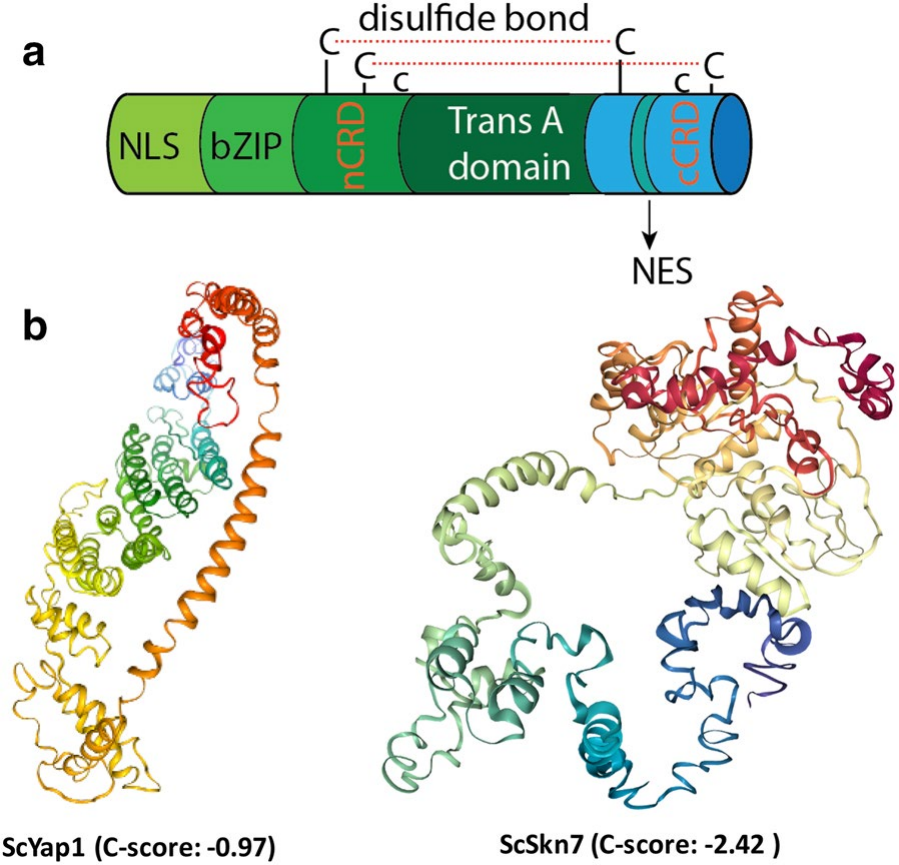
Structural model of yeast transcriptional factors that enable selective import/export of transcriptional factors across the nuclear membrane. **a** ScYap1 3-D structure contains the N-terminal nuclear import (NLS) signal that could be recognized by importin Pse1, and the C-terminal cysteine-rich domain (CRD) that sequestrates the leucine zipper nuclear export signal (ZIP-NES). Transcriptional activation domain is also indicated. **b** Structural model for ScYap1 and ScSkn7 predicted by bioinformatics software I-TASSER. C-score is a confidence score for estimating the quality of predicted models by I-TASSER [76]; a C-score of higher value signifies a model with a high confidence and vice versa

Using reactive oxygen species (ROS) response as an example, we may better understand how the importin and exportin control the nuclear transport of transcriptionally-active Yap1 and Skn7 (Fig. 4). Pse1 (Protein secretion enhancer) interacts with the nuclear pore complex and acts as the nuclear import receptor for many TFs including Yap1 [57]. Classical and arginine/glycine-rich NLSs are recognized by Pse1 [58]. Crm1 (Chromosome region maintenance factor) acts as the receptor for the leucine-rich nuclear export signal (LR-NES) involved in the export of proteins, RNAs, and ribosomal subunits from the nucleus [59]. In case of yeast AP-1 transcription factor Yap1, the accumulation of the oxidized Yap1 in the nucleus triggers the expression of anti-oxidative genes [60]. Both the NLS and basic leucine zipper nuclear export signal (bZIP-NES) are present in Yap1 (Fig. 4a), which determines the import/export of Yap1 across the nuclear envelope [61–63]. Under normal conditions, both NLS and NES are active. Yap1p is imported in and exported out of the nucleus via Pse1p and Crm1p, respectively. Two N-terminal amino acid regions (5-16 and 50-59) of Yap1p are crucial to the NLS activity. While NES sequence of Yap1 is embedded in the C-terminal cysteine rich domain (CRD) of Yap1p. Under oxidative conditions, oxidation of specific cysteine residues of CRD results in the formation of disulfide bonds and the masking or sequestration of NES (Fig. 4a), thus abolishing nuclear export by CRM1/exportin [62–64]. In a word, the oxidative states of the cell control the selective partition and subcellular localization of Yap1. Interestingly, both NLS and Pse1p are not affected by oxidative conditions.

With this knowledge, we may combine the NLS, effector-binding domain and b-ZIP NES sequence to design and engineer novel MRTFs that could be recognized by Pse1 or Crm1 (Fig. 4a, b). This nuclear transport mechanism provides us a structural guideline how importin or exportin could be harnessed to selectively control and partition transcriptionally-active MRTFs to improve the sensor activity. Except for enhancement of the import of TFs into nucleus, the other strategy is to prevent the exportation of small molecules or TFs out of the nucleus. It can be achieved by down-regulation or deletion of the gene for specific efflux pump or the genes encoding exportin.

## MRTF applications and future perspectives toward intelligent biomanufacturing

Genetically-encoded sensor-regulator system has proven as the efficient way to optimize cell metabolism and improve chemical manufacturing [16]. As an excellent tool to combat the metabolic heterogeneity, MRTF-based biosensor transduces an internal cellular signal to a transcriptional output and drive the expression of the designed genetic/biomolecular circuits to compensate the activity loss of the engineered biosystem [65]. The source of metabolic heterogeneity arises from a multitude of factors, including nutrients starvation, gene/protein expression burden, accumulation of toxic metabolites and environmental stress (ROS, osmotic pressure, heat shock) et al. The integration of MRTF with various feedback genetic circuits and biocatalytic function pave the ways for us to engineer efficient microbial cell factory with improved productivities and process economics [66].

**Fig. 5:**
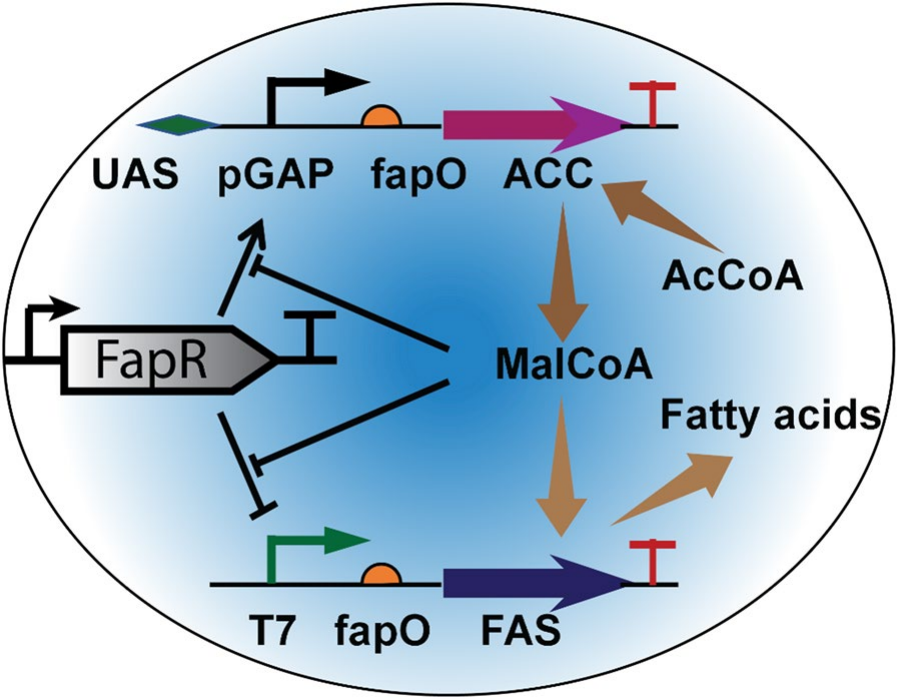
A classical malonyl-CoA switch to dynamically regulate fatty acids biosynthesis. FapR activates pGAP promoter which controls the transcription of the malonyl-CoA (Mal-CoA) source pathway (ACC) to provide malonyl-CoA; at the same time, FapR represses T7 promoter which controls the expression of the malonyl-CoA sink pathway (FAS) to consume malonyl-CoA. The activation of FapR to pGAP promoter depends on the upstream activation sequence (UAS); the repression of FapR to T7 promoter depends on the fapO sites (operator). High level of malonyl-CoA tunes down the expression of ACC, but tunes up the expression of FAS; low level of malonyl-CoA tunes up the expression of ACC, but tunes down the expression of FAS

MRTF-based biosensors have been applied in various areas including high throughput screening of desired strains [34, 49], dynamic control of biosynthetic pathway [9, 16, 65, 67], adaptive evolution [33], detection of pollutant or toxic compound [68–70] et al. An excellent example of dynamic pathway regulation has been summarized in Fig. 5. Reporter gene can also be swapped with a dosage-sensitive gene (e.g. *ACT1*, *CDC14* or *TPK2*) for screening of strains with desired characteristics [34], or with an auxotrophic marker gene (e.g. *HIS3*) for selection of histidine auxotrophic strains in the presence of small metabolite [26]. This will create a sensor-regulator system for adaptive metabolic control that is critical to achieve intelligent biomanufacturing. Under this scenario, when the sensor is used to control the expression of a growth-related gene (i.e. an auxotrophic marker *Leu2* or *HIS3*), this sensor-regulator system provides the means to connect a metabolite (i.e. end product of a pathway or an intermediary metabolite) with the growth fitness of the engineered cell. Engineering competitive growth advantage will allow metabolic engineers to develop growth-based screening strategies and selectively enrich the desired phenotype without referring to tedious analytical procedures.

Except for engineering various TF-based biosensors to expand the sensing capability, the definition of “reporter” is also greatly expanded. Very recently, a novel allosteric TF-based nicked DNA template-assisted signal transduction system (aTF-NAST) has been developed to transduce the signal of small metabolite via various conventional DNA detection technologies [71]. Briefly, the authors demonstrate that a single nick inside the TF binding site (TFBS) does not affect its function but can be recognized by T4 ligase. In the absence of effector molecule, the MRTF binds to the nick site, thus blocking the repair by T4 ligase. However, in the presence of effector molecule, TF will change its confirmation and fall off from the nicked TFBS, thus T4 DNA ligase can repair the nick [71]. Theoretically, such competitive effect between MRTF and T4 ligase enables the transduction of signals from any type of small metabolites into robust and sensitive DNA signals that could be easily detected in any in vitro system.

Eukaryotic cellular metabolism is complex and carried out by many genes with different levels of controls. Most of investigations focus on the construction of biosensor for the detection of a single small metabolite with improved abilities. As the progress of synthetic biology and protein evolution, fine-tuning the pool size of multiple metabolites via orthogonal TF-based biosensor becomes possible [72]. In another direction, optogenetic control provides a convenient way to interface in vivo gene expression with in vitro light signal [73, 74], ideally, this would enable the design of reprogrammable and predictable gene expression systems for intelligent biomanufacturing and smart therapeutics. Furthermore, integrating MRTF sensors with microbial communities presents tremendous opportunity to engineer cooperative phenotype or achieve dynamic population control in synthetic microbial consortia [75]. Computational and experimental approach will be critical to understand and unravel the design principles of the population-based sensor-actuator system or cross-feeding mechanisms that will lead to intelligent biosystems for various applications.

## Conclusions

MRTF consists of both a DNA-binding domain (DBD) and an effector-binding domain (EBD). This molecular architecture confers cell the ability to sense environmental signals and small molecules, and transduce the effector-binding activity to the DNA-binding activity. As an endeavor to engineer intelligent biomanufacturing systems, MRTFs are promising tools to translate a small-molecule sensing capability to a transcriptional output. Indeed, recent metabolic engineering advances have demonstrated that MRTFs are indispensable tools to dynamically allocate cellular resources and optimally control pathway expression (Fig. 5). Most of characterized MRTFs are derived from bacteria. The success of translating bacteria-derived TFs to sense small molecules in eukaryotic cells depends on whether the engineered MRTFs are compatible with chassis-specific biological parts. To successfully implement and engineer such molecular sensors, one should consider the intrinsic genetic differences between bacteria and eukaryotic cells in terms of replication, transcription, translation, post-translational modification and nuclear transport et al.

Due to the cellular complexity of eukaryotic cells, the choice of reporter gene determines the background noise and the approach of how to characterize the biosensors. Because of the simplicity, high sensitivity and low background noise, ATP-independent luciferase (NanoLuc) is a promising reporter to characterize eukaryotic transcriptional systems. Mutagenesis, directed evolution and computational tools are important to engineer novel DNA-binding and effector-binding domains. Promoter strength, the position and number of activator or repressor-biding sites are critical to tune the dynamic response, the sensitivity and specificity of engineered sensors. Repression-based transcriptional regulation is rare in eukaryotic cells, a transcriptional activation domain is generally required to translate a bacteria repressor to a MRTF sensor in eukaryotic cells. To enhance the sensitivity and the detection limit, one should always consider using a minimal promoter containing only the core TF binding sites and the TATA box. Nuclear import and export of transcriptionally active MRTFs plays a critical role in regulating the sensor activity. The molecular underpinnings and structure of nuclear localization signal (NLS), nuclear export sequence (NES) and transcriptional activation domain were exemplified with a yeast-derived ROS sensor Yap1 and Skn7. Rational design of novel MRTF sensors is possible via computational approach and protein evolution.

Taken together, MRTFs have proven as powerful tools to control cell metabolism and deliver robust microbial cell factories. Integrating sensor-regulator systems to build biological devices that sense, respond, and compensate the metabolic activity of the engineered system is a critical step to achieve intelligent biomanufacturing. It is anticipated that novel genetic regulatory tools, including optogenetic regulation, fast dynamics split-biosensors, and genome-editing tools would facilitate the development of more sensitive molecular control device. Uncovering the design principles underlying MRTF and engineering predictable sensor-regulator system would help us encode decision-making function into living cell factories and improve the cost-competitiveness of industrial biomanufacturing beyond conventional process engineering strategies.
